# Diagnostic accuracy and potential triage utility of the Shetty test in foot and ankle trauma: a cross-sectional study

**DOI:** 10.55730/1300-0144.6041

**Published:** 2025-06-14

**Authors:** Burcu DOĞAN, Seval KOMUT, Erdal KOMUT, Nezih KAVAK, Osmancan GÜNEŞ, Anılcan Tahsin KARAHAN

**Affiliations:** 1 Department of Emergency, Faculty of Medicine, University of Health Sciences, Ankara, Turkiye; 2 Department of Emergency, Faculty of Medicine, Hitit University, Çorum, Turkiye; 3 Department of Radiology, Faculty of Medicine, Hitit University, Çorum, Turkiye; 4 Department of Emergency, Faculty of Medicine, Necmettin Erbakan University, Konya, Turkiye

**Keywords:** Shetty test, clinical decision rule, emergency department, foot and ankle trauma, triage

## Abstract

**Background/aim:**

Foot and ankle trauma represents a common reason for emergency department visits. While the majority of cases involve soft tissue injuries, radiographic imaging is frequently overutilized due to concerns about missed fractures, leading to increased costs and emergency department crowding. The Shetty test, a recently introduced clinical decision rule, may serve as a simpler alternative to established tools such as the Ottawa ankle rules. This study aimed to assess the diagnostic accuracy of the Shetty test and its potential role as a supportive tool within existing triage systems for patients presenting with foot and ankle trauma.

**Materials and methods:**

In this cross-sectional study, 229 adult patients with isolated foot or ankle trauma were evaluated in the emergency department. All participants underwent the Shetty test and standard radiographic imaging. The Shetty test was performed by trained emergency physicians prior to imaging; a positive result was defined as an inability to apply downward pressure due to pain. Diagnostic accuracy metrics—including sensitivity, specificity, positive predictive value, and negative predictive value—were calculated using radiographic findings as the reference standard.

**Results:**

Fractures were identified in 25.3% of cases. The Shetty test demonstrated a sensitivity of 77.6%, specificity of 60.8%, positive predictive value of 40.2%, and a high negative predictive value of 88.9%. Among patients with confirmed fractures, 77.6% had a positive test result. The test performed best in ruling out displaced and incomplete fractures, and results showed significant correlation with both physical findings and imaging outcomes.

**Conclusion:**

The Shetty test exhibited moderate sensitivity and specificity, alongside a high negative predictive value, supporting its use as a reliable rule-out tool for foot and ankle fractures. Its simplicity, ease of application, and diagnostic potential make it a promising triage adjunct to optimize emergency department resource use. Prospective multicenter validation is warranted before broad clinical adoption.

## 1. Introduction

Foot and ankle trauma is among the most common reasons for emergency department (ED) visits, encompassing a wide range of injuries from minor soft tissue sprains to complex fractures [1–3]. Although soft tissue injuries account for the majority of cases, fractures are identified in approximately 10%–15% of presentations [4–6].

The standard diagnostic approach typically includes clinical evaluation followed by radiographic imaging. However, concerns about missed diagnoses and medicolegal repercussions often lead to a defensive medical strategy, with imaging frequently requested before a thorough physical examination is conducted [5,7,8]. This practice contributes to unnecessary radiation exposure, increased healthcare costs, ED overcrowding, and prolonged patient stays [9].

Given the relatively low prevalence of fractures despite high imaging rates, clinical decision rules (CDRs) have become increasingly important in guiding radiographic decision-making. The Ottawa ankle rules (OAR) are the most extensively validated and widely adopted CDR for foot and ankle trauma, offering excellent sensitivity but limited specificity [10,11–13]. The Shetty test (ST), a more recently proposed CDR, has demonstrated both high sensitivity and specificity in preliminary studies and may serve as a simpler, more practical alternative to the OAR in busy ED settings [14–16].

Despite its potential, the ST remains underrepresented in the literature, with limited data on its diagnostic accuracy and clinical utility. This study aims to evaluate the diagnostic performance of the ST in predicting the need for radiographic imaging in patients presenting to the ED with foot and ankle trauma, and to further clarify its potential role in enhancing clinical decision-making.

## 2. Materials and methods

### 2.1. Study design and ethical approval

This observational, cross-sectional study was conducted in accordance with the Declaration of Helsinki and received approval from the Hitit University Noninterventional Research Ethics Committee (approval no: 2022–23; date: 11.04.2022).

### 2.2. Sample size justification

A priori power analysis was conducted using G*Power 3.1 software (Heinrich Heine University, Düsseldorf, Germany) to determine the minimum required sample size for detecting significant differences between two independent groups using a two-tailed t-test. The analysis assumed a medium effect size (d = 0.5), an alpha level of 0.05, and a desired power of 0.95. Based on these parameters, the required sample size was calculated to be 210 participants. To account for an anticipated 10%–20% rate of missing or unusable data, the target enrollment was set at approximately 229 patients.

### 2.3. Participants

A total of 229 consecutive adult patients presenting to the ED minor trauma unit with isolated foot or ankle trauma were enrolled. Written informed consent was obtained from all participants. Exclusion criteria included age under 18 years, pregnancy, multiple trauma, open fractures, gross deformities suggestive of fracture, physical or mental conditions impairing cooperation, recent use of analgesics or antiinflammatory drugs, lack of trauma history, and recent foot or ankle surgery.

### 2.4. Data collection

A structured data collection form was used to record demographic information, mechanism of injury, pain localization, and physical examination findings. Radiographic evaluation (anteroposterior (AP) and lateral foot/ankle X-rays) was performed for all patients and interpreted by a single, blinded researcher (radiologist) who was not involved in test administration.

### 2.5. The Shetty test protocol

Physicians performing the ST had a minimum of 6 months’ experience in the ED and underwent standardized training before study initiation. Each physician completed a minimum of 20 supervised applications of the test prior to data collection.

The test was performed with the patient seated, legs hanging freely. The patient was instructed to place the sole of the affected foot onto the examiner’s dominant palm and apply downward pressure, simulating a weight-bearing action. A positive test result was defined as the inability to apply force due to pain, suggesting a potential fracture. A negative result was defined as the ability to apply force without significant pain, indicating a low likelihood of fracture [14,15].

### 2.6. Statistical Analysis

Statistical analyses were performed using SPSS software (Version 22.0; SPSS Inc., Chicago, IL, USA). Descriptive statistics were presented as frequencies and percentages for categorical variables, and as mean ± standard deviation for continuous variables. The Kolmogorov-Smirnov test was used to assess the normality of distribution. Numerical variables were compared using the Student’s t-test, while categorical variables were analyzed using the Chi-square test or Fisher’s exact test, as appropriate. Diagnostic accuracy measures—including sensitivity, specificity, positive predictive value, and negative predictive value—were calculated. A p-value of <0.05 was considered statistically significant.

## 3. Results

### 3.1. Patient characteristics

A total of 229 patients were included in the study, comprising 49.8% female and 50.2% male participants, with a mean age of 38.5 ± 13.7 years (range: 18–79). Pain localization was reported as follows: ankle pain in 63.8% of patients, foot pain in 35.4%, and combined foot and ankle pain in 0.9%. The most common mechanisms of trauma were sprain (55.9%), fall (18.3%), stubbing (11.8%), direct impact (7.4%), and combined fall and sprain (6.6%).

Physical examination findings included localized tenderness in 39.3% of patients, swelling in 9.2%, ecchymosis in 4.4%, and combinations thereof. Radiographic imaging identified fractures in 58 patients (25.3%): 6.6% were displaced, 14.4% nondisplaced, and 4.4% incomplete. No radiographic evidence of fracture was observed in 74.7% of cases.

### 3.2. Shetty test results and group comparisons

The ST was positive in 112 patients (48.9%) and negative in 117 patients (51.1%). Comparisons of socio-demographic and clinical characteristics based on ST results are presented in Tables 1 and 2. No statistically significant differences were observed between the groups in terms of age (p = 0.716), sex (p = 0.166), pain localization (p = 0.632), or trauma mechanism (p = 0.272).

In contrast, physical examination and radiographic findings were significantly associated with ST results (p = 0.018 and p < 0.001, respectively). Among patients with positive physical examination findings, 108 patients (51.2%) also had a positive ST result. Additionally, 45 patients (77.6%) of those with radiographically confirmed fractures tested positive on the ST.

### 3.3. Diagnostic accuracy of the Shetty test

The distribution of the ST results according to physical examination and radiographic findings is summarized in Table 3. The ST was positive in 13 patients (86.7%) with displaced fractures, in 23 patients (69.7%) with nondisplaced fractures, and in 9 patients (90.0%) with incomplete fractures. Among patients without radiographic fractures, 67 patients (39.2%) had a false-positive ST result.

Crosstab analysis and diagnostic accuracy measures are detailed in Tables 4A and 4B. For predicting the need for radiographic imaging, the ST demonstrated a sensitivity of 77.6% (95% CI, 64.4–87.1), specificity of 60.8% (95% CI, 53.1–68.1), positive predictive value (PPV) of 40.2% (95% CI, 31.2–49.9), and negative predictive value (NPV) of 88.9% (95% CI, 81.4–93.7).

## 4. Discussion

Soft tissue injuries are the most frequently encountered musculoskeletal conditions among patients presenting to EDs with foot and ankle trauma. These cases often lead to excessive imaging, unnecessary radiation exposure, increased healthcare costs, and ED overcrowding [1,6–9]. In this context, our study demonstrated that the ST yields a high NPV of 88.9%, suggesting its potential utility in safely excluding fractures without the immediate need for radiographic evaluation. A negative ST result was strongly associated with a low likelihood of fracture, highlighting its value in minimizing unnecessary imaging and supporting more efficient, evidence-based decision-making in EDs.

In our analysis, the ST demonstrated a sensitivity of 77.6%, specificity of 60.8%, PPV of 40.2%, and NPV of 88.9%. The notably high NPV underscores the strength of the ST in reliably ruling out fractures, particularly in low-risk cases. However, the relatively low PPV indicates limited ability to confirm fractures, reinforcing its role as a rule-out rather than rule-in tool in clinical assessment.

The existing literature on the ST remains limited, with few studies having evaluated its diagnostic performance in foot and ankle trauma [7,14–16]. The present study contributes to this body of evidence by assessing the ST in a real-world ED setting, thereby enhancing its clinical relevance and applicability in routine practice.

Prior investigations have reported varying diagnostic performance for the ST. Shetty et al. initially reported a sensitivity of 100% and specificity of 91% [14], while Jimenez et al. observed similar sensitivity (100%) but slightly higher specificity (95.6%) [15]. Conversely, a domestic study reported lower sensitivity (51.4%) but relatively high specificity (85.9%) [7]. The diagnostic metrics in the present study fall within this spectrum, indicating intermediate performance and underscoring the influence of population characteristics, methodology, and clinical context on test outcomes.

This variability may stem from differences in study design, patient demographics, and injury spectrum. Earlier studies often involved smaller, more homogeneous cohorts, which may partly explain the higher sensitivity and specificity observed [14,15]. Other influencing factors include clinician expertise, patient cooperation, and consistency in test administration. The broader and more heterogeneous population in our study likely accounts for the intermediate results and enhances the external validity of our findings.

The OAR, developed by Stiell, remains the most widely validated and internationally adopted CDR for foot and ankle trauma [11]. The OAR has proven effective in reducing unnecessary radiographic imaging, shortening ED stays, and lowering healthcare costs [12]. Two major studies reported a mean sensitivity of 99.4% with a specificity of 35.3%, emphasizing the rule’s focus on avoiding missed fractures rather than minimizing imaging use [13,17]. A four-phase validation study showed reductions in radiographic use by 14% for foot injuries and 28% for ankle injuries, along with an average 36 min reduction in ED stay [11]. More recent research reports imaging reductions of up to 72.5%, reinforcing the OAR’s utility as a safety-oriented screening tool [18–20].

Despite its established advantages, the OAR has certain practical limitations. Accurate application requires palpation of specific anatomical landmarks, which may be challenging in patients with significant pain or soft tissue swelling. Furthermore, the requirement for patients to ambulate four steps may be infeasible in those with severe pain or suspected fractures [5,8,12].

In contrast, the ST offers several practical benefits. It is easier to administer, does not rely on precise palpation, and is suitable for patients who are unable to bear weight due to pain or deformity [7,14]. Additionally, the high NPV (88.9%) supports its reliability in ruling out fractures among low-risk patients. Our findings suggest that the ST could serve as a valuable adjunct to existing decision-making protocols, offering a simpler and more efficient alternative for initial assessment. Nevertheless, the lower sensitivity observed in this study compared to the OAR suggests that the ST should not yet be viewed as a standalone diagnostic tool. Further prospective research is necessary to validate its role in broader clinical workflows.

Given its high NPV, the ST may also support ED triage by facilitating early identification of low-risk patients. Rather than altering formal triage categories, the ST could assist in stratifying patients within existing levels—particularly in intermediate zones such as “yellow”—by identifying those with minor orthopedic injuries who can safely tolerate longer wait times. This targeted deprioritization may enable more efficient resource allocation and allow higher-acuity presentations to receive timely care without compromising safety. This approach represents a shift from traditional sensitivity-driven decision-making toward a workflow-conscious triage strategy. If validated in prospective, real-world settings, the integration of the ST into triage protocols could improve departmental efficiency while maintaining diagnostic rigor.

### 4.1. Limitations

This study has several limitations that should be acknowledged. First, its single-center design may limit the generalizability of the findings to broader populations or different clinical settings. Second, only standard anteroposterior and lateral radiographs were utilized; the absence of adjunctive imaging modalities such as mortise or oblique views may have resulted in underdiagnosis of subtle fractures. Third, foot dominance and individual pain thresholds were not considered, both of which could influence test performance through variations in neuromuscular loading and subjective pain perception.

Fourth, the OAR were not applied prospectively or systematically documented in the study population, precluding direct comparative validation. Future research should address this gap by evaluating both the ST and OAR within the same patient cohort using a prospective design. Additionally, multicenter studies incorporating broader patient populations, more comprehensive imaging protocols, and stratification by relevant clinical variables are warranted to enhance external validity and diagnostic precision.

Moreover, each patient in this study was evaluated by a single physician as part of routine clinical care. While this approach reflects real-world practice, it did not allow for assessment of interrater reliability, which may be considered a methodological limitation. The observational nature of the study and concerns over workflow disruption precluded the inclusion of a second examiner.

Finally, children under the age of five and patients unable to follow instructions due to pain, altered mental status, or cognitive impairment were excluded, as the ST requires active participation and comprehension. Although necessary to preserve test validity, this exclusion limits the generalizability of the findings to these subpopulations.

Future research should address these limitations by incorporating multicenter, prospective randomized trials, including pediatric patient groups, and evaluating the applicability of the ST in healthcare systems with limited access to imaging resources.

## 5. Conclusion

The ST demonstrated a high NPV and moderate specificity, supporting its potential role in reducing unnecessary radiographic imaging and related healthcare costs, particularly in low-risk cases of foot and ankle trauma. However, due to the variability in diagnostic accuracy reported across studies, widespread adoption cannot yet be recommended. Prospective, multicenter studies are essential to validate its diagnostic performance and assess its generalizability across diverse clinical settings. Future research should also compare the real-world utility of the ST with established clinical decision rules, such as the OAR, to better define its place within emergency care protocols.

## Figures and Tables

**Table 1:** Comparison of demographic variables based on Shetty test results.

Variable	Shetty test positive (n = 112)	Shetty test negative (n = 117)	p-value
**Age, mean ± SD**	38.13 ± 13.53	38.79 ± 13.95	0.716^a^
**Sex**			0.166^b^
Female	61 (53.5%)	53 (46.5%)	
Male	51 (44.3%)	64 (55.7%)	

a Student’s *t*-test;

b Pearson chi-square test were used as appropriate.

**Table 2:** Comparison of clinical characteristics according to Shetty test results.

Variable	Shetty test positive (n = 112)	Shetty test negative (n = 117)	p-value
**Pain site**			0.632^a^
Foot	40 (49.4%)	41 (50.6%)	
Ankle	72 (49.3%)	74 (50.7%)	
Both	0 (0%)	2 (100%)	
**Mechanism of trauma**			0.272^b^
Fall	27 (64.3%)	15 (35.7%)	
Sprain	59 (46.1%)	69 (53.9%)	
Direct impact	8 (47.1%)	9 (52.9%)	
Stubbing	11 (40.7%)	16 (59.3%)	
Fall + sprain	7 (46.7%)	8 (53.3%)	
**Physical examination finding**			**0.018** b
Absent	4 (22.2%)	14 (77.8%)	
Present	108 (51.2%)	103 (48.8%)	
**X-ray findings**			**<0.001** b
No fracture	67 (39.2%)	104 (60.8%)	
Fracture present	45 (77.6%)	13 (22.4%)	

a Fisher’s exact test;

b Pearson chi-square test were used as appropriate.

**Table 3:** Relationship between Shetty test results and clinical/radiographic findings.

Clinical findings	Shetty test positive (n = 112)	Shetty test negative (n = 117)	p-value
**Physical examination**			**0.045** a
Tenderness	41 (45.6%)	49 (54.4%)	
Swelling	11 (52.4%)	10 (47.6%)	
Ecchymosis	4 (40.0%)	6 (60.0%)	
Temperature change	1 (33.3%)	2 (66.7%)	
Tenderness and swelling	40 (59.7%)	27 (40.3%)	
Tenderness and ecchymosis	1 (25.0%)	3 (75.0%)	
Tenderness, swelling and ecchymosis	10 (71.4%)	4 (28.6%)	
Tenderness, swelling and temperature change	0 (0.0%)	2 (100.0%)	
No clinical finding	4 (22.2%)	14 (77.8%)	
**X-ray findings**			**<0.001** b
Displaced fracture	13 (86.7%)	2 (13.3%)	
Non-displaced fracture	23 (69.7%)	10 (30.3%)	
Incomplete fracture	9 (90.0%)	1 (10.0%)	
No fracture	67 (39.2%)	104 (60.8%)	

a Fisher’s exact test;

b Pearson chi-square test were used as appropriate.

**Table 4A:** Crosstabulation of Shetty test results and radiographic findings.

Shetty test result	Fracture present	No fracture	Total
**Positive**	45	67	112
**Negative**	13	104	117
**Total**	58	171	229

**Note:** Values indicate the number of patients.

**Table 4B:** Diagnostic performance of the Shetty test.

Metric	Value (95% CI)
**Sensitivity**	77.6% (64.4–87.1)
**Specificity**	60.8% (53.1–68.1)
**Positive predictive value (PPV)**	40.2% (31.2–49.9)
**Negative predictive value (NPV)**	88.9% (81.4–93.7)
